# Performance of FDA-Approved AI Algorithms in Detecting Acute Pulmonary Embolism on Computed Tomographic Pulmonary Angiography (CTPA): A Meta-Analysis of Real-World Retrospective Studies

**DOI:** 10.7759/cureus.94391

**Published:** 2025-10-12

**Authors:** Ethan X DePry, Viren Parmar, Saahas Rajendran, Victor Milev, Mukul Anand, Manik Anand, Sarabjeet Singh

**Affiliations:** 1 Medicine, Florida Atlantic University Charles E. Schmidt College of Medicine, Boca Raton, USA; 2 Statistics, University of Pennsylvania, Philadelphia, USA; 3 Research, Pepperdine University, Malibu, USA; 4 Cardiology, Bakersfield Heart Hospital, Bakersfield, USA

**Keywords:** acute pulmonary embolism, ai and machine learning, aidoc, artificial intelligence, ct pulmonary angiogram (ctpa), deep learning artificial intelligence, machine learning, pulmonary ct angiography, pulmonary embolism (pe)

## Abstract

Pulmonary embolism (PE) is a potentially fatal condition requiring prompt and accurate diagnosis. Computed tomographic pulmonary angiography (CTPA) is the gold standard for PE detection, but its interpretation is time-intensive and subject to human error. Recent advancements in artificial intelligence (AI), particularly in machine learning (ML) and deep learning (DL) algorithms, offer promising tools to enhance diagnostic efficiency and accuracy. This systematic review and meta-analysis evaluated the diagnostic performance of FDA-approved ML algorithms for detecting acute PE on CTPA. A comprehensive literature search across six databases identified six retrospective studies encompassing 9,102 CTPA scans, all of which assessed either the Aidoc or CINA-PE algorithms. Pooled sensitivity and specificity were 93% (95% CI: 88%-95%) and 98% (95% CI: 93%-100%), respectively, indicating high diagnostic accuracy across real-world datasets. All included studies demonstrated low risk of bias according to Quality Assessment of Diagnostic Accuracy Studies-2 (QUADAS-2) assessments. These findings support the integration of FDA-approved ML tools into radiological workflows as adjuncts to reduce diagnostic errors and improve triage. However, prospective trials are needed to assess their impact on clinical decision-making, workflow efficiency, cost-effectiveness, and patient outcomes.

## Introduction and background

Pulmonary embolism (PE) is a serious and often life-threatening condition that occurs due to a blockage of the pulmonary arteries, typically due to embolized clots, tissue, or air bubbles [1,2]. This obstruction may lead to rapid cardiovascular and respiratory compromise, making prompt diagnosis and intervention critical to reducing mortality [1,2]. The American College of Physicians recommends computed tomographic pulmonary angiography (CTPA) as the first-line diagnostic test for patients with a high pretest probability of PE, provided there are no contraindications to the use of radiographic contrast dye [3-5]. CTPA offers the advantage of rapid image acquisition and the ability to visualize both central and peripheral pulmonary arteries, making it an effective tool in emergency settings [4-6]. However, CTPA is not without limitations, as image quality may be affected by motion artifacts, suboptimal contrast bolus timing, and patient-specific factors such as renal impairment or allergy to iodinated contrast [5,6]. In addition, interpretation requires significant radiologist expertise, and interobserver variability can contribute to missed or equivocal diagnoses [3,6].

Despite being widely regarded as the gold standard for PE diagnosis, the use of CTPA may be limited by time and the need for radiologist expertise. The implementation of artificial intelligence (AI) into the clinical workflow has shown potential in enhancing the efficiency and accuracy of radiologists, especially in cases of pulmonary embolism [7,8]. AI also has potential in mitigating the growing demand for radiology services, acting as an assistant for radiologists who must manage a high volume of imaging studies while maintaining diagnostic accuracy [3,9,10].

A subtype of AI designed for pattern recognition, deep learning (DL) algorithms have begun to be implemented into medical imaging analysis and show promise in supporting radiology workflows [11-13]. DL algorithms trained on large CTPA datasets can detect patterns associated with PE [13,14]. Convolutional neural networks (CNNs) are a subtype of DL tailored for image analysis [14-16]. In the context of CTPA, CNNs can be trained to detect pulmonary emboli by learning from annotated datasets [14,16]. Recent studies have demonstrated the efficacy of CNNs in detecting PE with a high sensitivity, suggesting this tool’s utility for quickly ruling out PE in clinical practice [14,16].

While numerous experimental AI models have demonstrated strong performance in research settings, the clinical impact of such tools may be limited until they receive regulatory clearance. FDA-approved algorithms, unlike investigational models, undergo rigorous validation before being integrated into clinical workflows, ensuring that these diagnostic tools meet standards for reliability, transparency, and patient safety [17,18]. Studying these cleared algorithms is therefore particularly important, as they represent the subset of AI tools that clinicians can actually deploy in practice, offering direct relevance to patient care [17,18].

Current FDA-cleared and CE-marked DL algorithms include Aidoc and CINE-PE, both of which are DL-powered algorithms that are designed to assist radiologists by automatically detecting PE on CTPA scans and have shown efficacy in helping to prioritize cases for review and potential in reducing diagnostic delays [19,20]. This systematic review aims to evaluate the pooled efficacy of FDA-approved DL algorithms in the detection of PE on a per-case level.

## Review

Methods

Our systematic review and meta-analysis are reported according to the latest Preferred Reporting Items for Systematic Reviews and Meta-analyses 2020 (PRISMA 2020) guidelines [21]. A keyword search of PubMed, MEDLINE, Embase, SCOPUS, Cochrane, and Web of Science was conducted on October 29, 2024. Keywords related to AI were "deep learning,” "machine learning,” "artificial intelligence,” “CNN,” “convolutional neural networks,” “neural network,” and “neural networks.” Keywords related to pulmonary embolism and CTPA were “pulmonary embolism,” “PE,” “APE,” “CTPA,” “CT pulmonary angiograph,” and “computed tomography pulmonary angiography.” Inclusion criteria were peer-reviewed original studies that (1) evaluated an FDA-approved DL model for the detection of APE on CTPA, (2) reported specificity and sensitivity as outcome measures, and (3) were published in English. We excluded non-DL articles, studies on multimodal data, proof-of-concept studies, and studies reporting on non-FDA-approved ML algorithms.

Two authors (ED and VM) independently screened the titles and abstracts of the studies to determine if the studies met the inclusion criteria using the Covidence systematic review management tool. If the title met the inclusion criteria but the abstract did not include the name of the ML algorithm used and/or FDA approval status, then the full-text article was reviewed. In cases of discrepancy, a third reviewer (VP) was used to resolve any disagreements. In cases where discrepancies in data extraction occurred, the reviewers compared results line-by-line and discussed differences until agreement was reached; if disagreement persisted, the third reviewer adjudicated.

Two reviewers (ED and VM) extracted data independently from the articles. The data extracted included the publication year, study design, ML model tested, dataset that the algorithm was tested on, number of CTPAs included in the study, reference standards used, and evaluation metrics. Evaluation metrics that were extracted were true positives, true negatives, false positives, false negatives, sensitivity, specificity, positive predictive value (PPV), negative predictive value (NPV), and/or accuracy. In cases where discrepancies in data extraction occurred, the reviewers compared results line-by-line and discussed differences until agreement was reached; if disagreement persisted, the third reviewer adjudicated.

Two reviewers independently used the Quality Assessment of Diagnostic Accuracy Studies-2 (QUADAS-2) to assess risk of bias in the selected studies [22]. The QUADAS-2 tool assesses risk across the domains of patient selection, index test, reference standard, and flow and timing. Studies were classified as having a low or high risk of bias across each of these domains. Conflicts were resolved through structured discussion, with the third reviewer consulted when consensus could not be achieved.

We performed a bivariate random‐effects meta‐analysis of sensitivity and specificity using the Reitsma approach (logit‐transformed outcomes) with restricted maximum‑likelihood estimation as implemented in the metafor R package. Between‐study variability and the covariance between sensitivity and specificity were modeled via an unstructured variance-covariance matrix, and summary receiver‑operator characteristic (SROC) curves, forest plots, and 95% confidence regions were generated using the mada package in R [23]. Due to the small number of included studies (fewer than 10), formal assessment of publication bias (e.g., funnel plot asymmetry or Egger’s regression test) was not performed [24].

Study selection and characteristics

**Figure 1:**
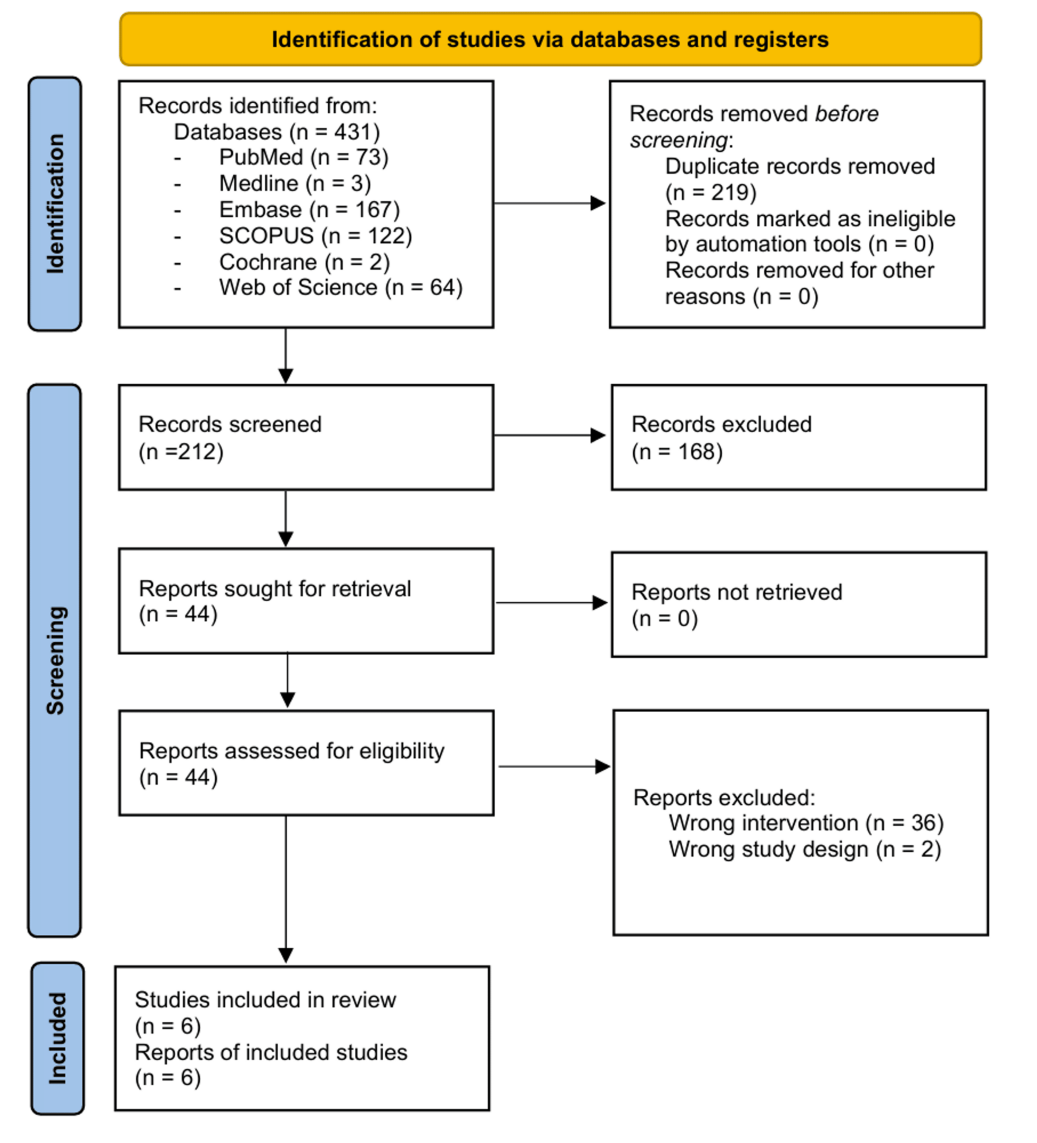
PRISMA 2020 flow diagram

A total of 431 studies from PubMed, MEDLINE, Embase, SCOPUS, Cochrane, and Web of Science were imported into Covidence, with 219 duplicates being removed. A total of 212 studies were screened, with a total of 168 being deemed irrelevant and 44 full-text studies assessed for eligibility. In total, six studies met the inclusion criteria (Figure 1). Characteristics of these studies are summarized in Table 1. The studies were published between 2020 and 2024, and all studies had a retrospective design. Most of the studies used the original radiology reports on the CTPA scans as the ground truth. Five of the studies evaluated the Aidoc DL algorithm, while one study evaluated the CINA-PE DL algorithm.

**Table 1:** Characteristics of included studies This table gives a summary of the articles in our literature review that assessed FDA-approved deep learning algorithms for pulmonary embolism detection on CT pulmonary angiography. Sources: Weikert et al., 2020 [19], Schmeulling et al., 2022 [25], Cheikh et al., 2022 [26], Zaazoue et al., 2022 [27], Languis-Wiffen et al., 2023 [28], and Ayobi et al., 2024 [20].

Author	Study Design	Database Type	Dataset Size (N)	Model Type	Ground Truth	Sensitivity	Specificity
Weikert et al., 2020 [19]	Retrospective	Institutional dataset	1465	Aidoc DL Algorithm	Original radiology reports	92.7 [88.5-95.7]	95.5 [94.2-96.6]
Schmeulling et al., 2021 [25]	Retrospective	Institutional dataset	411	Aidoc DL Algorithm	Original radiology reports	79.6 [70.8-87.2]	95.0 [92.0-97.1]
Cheikh et al., 2022 [26]	Retrospective	Multicenter institutional dataset (Cohort 2019)	1202	Aidoc DL Algorithm	Senior radiologist + AI expert	92.6 [87.9-95.9]	95.8 [94.3-96.9]
Zaazoue et al., 2023 [27]	Retrospective	Institutional dataset	1504	Aidoc DL Algorithm	Original radiology reports	93.2 [90.6-95.2]	99.6 [98.9-99.9]
Languis-Wiffen et al., 2023 [28]	Retrospective	Institutional dataset	3316	Aidoc DL Algorithm	Blinded consensus from two radiologists	96.8 [95.5-98.1]	99.9 [99.8-100.0]
Ayobi et al., 2024 [20]	Retrospective	Public dataset	1204	CINA-PE v1.0.5	Blinded consensus from two radiologists	93.9 [89.3-96.9]	94.8 [93.3-96.1]

Descriptive summary of results

Across the six included studies, FDA-approved DL algorithms consistently demonstrated strong diagnostic performance for detecting PE on CTPA. Reported sensitivities generally exceeded 90% [19,20,26-28], while specificities frequently surpassed 95% [19,20,26-28]. In fact, some studies reported near-perfect diagnostic accuracy, with one analysis showing a specificity of 99.9% [28]. These findings collectively highlight the algorithms’ ability to maintain both high sensitivity, which is important for reducing missed diagnoses, and high specificity, which is important for minimizing false positives and unnecessary downstream interventions. However, substantial heterogeneity was observed across studies, with I² = 87.1% for sensitivity and I² = 98.5% for specificity, indicating high variability in diagnostic performance between datasets. While the pooled accuracy is robust, differences in patient populations, imaging quality, annotation standards, and AI deployment workflows likely contributed to variable results.

A notable trend across the literature was the robustness of algorithm performance in diverse clinical contexts. For example, Cheikh et al. demonstrated that diagnostic accuracy was preserved even in cases with poor to moderate CTPA injection quality [26], while Zaazoue et al. found that performance was unaffected by COVID-19-related parenchymal disease [27]. These results underscore the adaptability of ML tools across technically challenging and clinically variable scenarios.

Beyond accuracy, several studies showed that ML systems were able to identify emboli missed by radiologists. The CINA-PE algorithm, for instance, detected 76% of pulmonary emboli that had initially been overlooked, reducing the miss rate from 15.6% to 3.8% [20]. Languis-Wiffen et al. also similarly concluded that missed positive findings on CTPA could be prevented through AI implementation [28]. Together, these findings emphasize the potential of ML algorithms to serve as effective safety nets in clinical workflows.

While diagnostic accuracy was consistently high, the impact on clinical workflow was less clear. One study examining downstream effects found no significant improvement in report communication times, initiation of anticoagulation, or emergency department throughput following algorithm integration [25]. This suggests that the ultimate benefit of ML adoption may depend not only on diagnostic performance but also on integration strategies, radiologist acceptance, and institutional workflow design.

**Figure 2:**
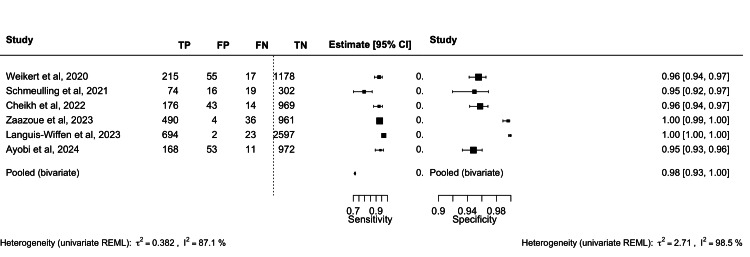
Combined forest plot of included studies Sources: Weikert et al., 2020 [19], Schmeulling et al., 2022 [25], Cheikh et al., 2022 [26], Zaazoue et al., 2022 [27], Languis-Wiffen et al., 2023 [28], and Ayobi et al., 2024 [20]. REML: Restricted maximum likelihood; TP: True positive; TN: True negative; FP: False positive; FN: False negative.

**Figure 3:**
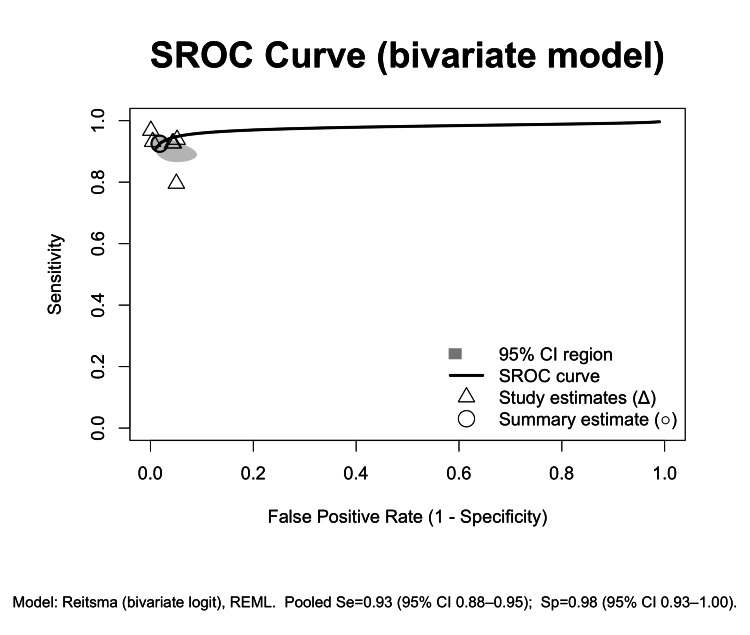
Bivariate summary ROC curve REML: Restricted maximum likelihood; SROC: Summary receiver operating characteristic.

The results of the bivariate random‐effects meta‐analysis of sensitivity and specificity yielded a pooled sensitivity of 92.5% and specificity of 98.2% for these studies (Figures 2, 3).

Quality assessment

**Figure 4:**
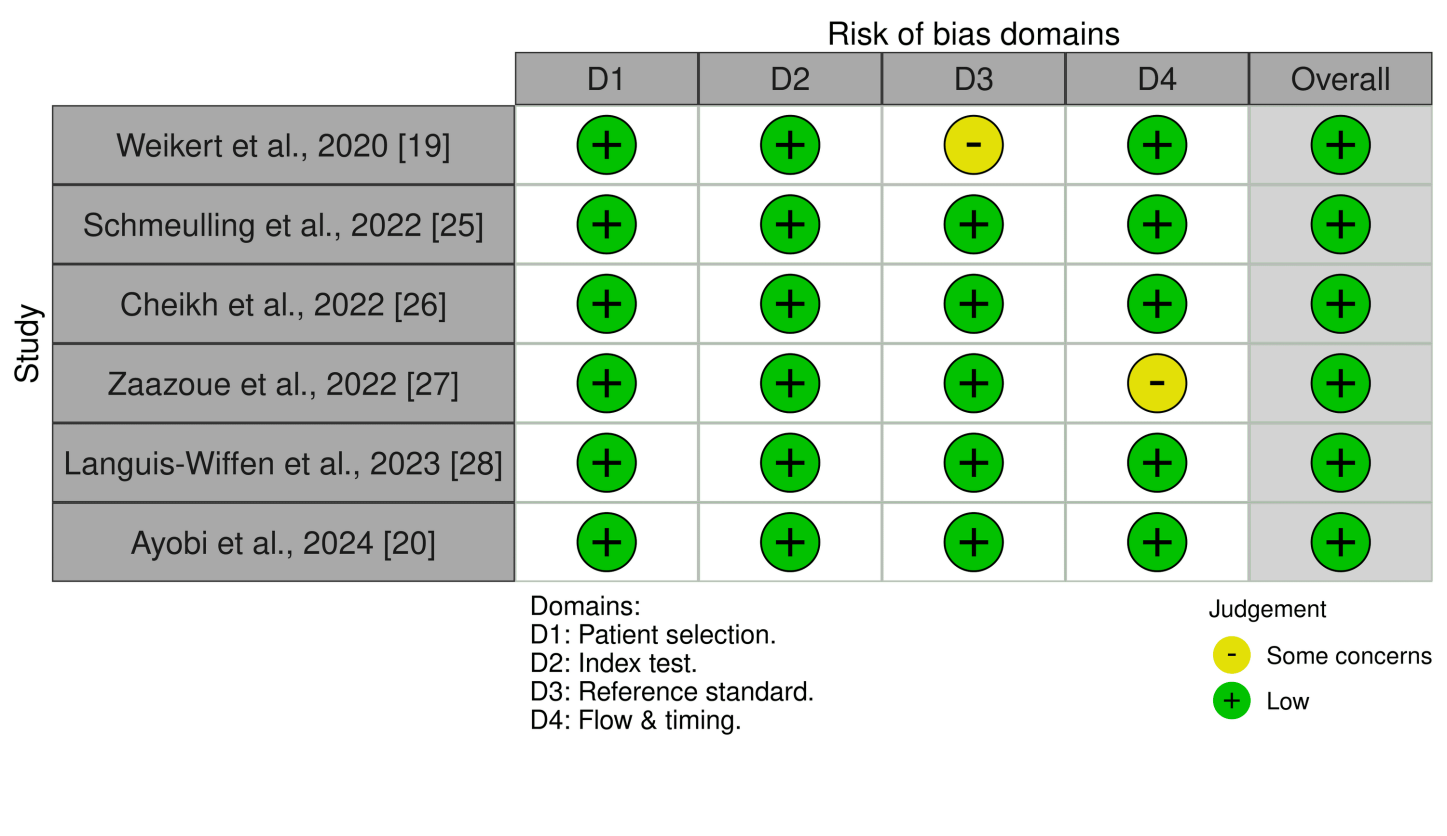
QUADAS quality assessment Sources: Weikert et al., 2020 [19], Schmeulling et al., 2022 [25], Cheikh et al., 2022 [26], Zaazoue et al., 2022 [27], Languis-Wiffen et al., 2023 [28], and Ayobi et al., 2024 [20]. QUADAS: Quality Assessment of Diagnostic Accuracy Studies.

Figure 4 presents an overview of the quality assessment of the included studies (N = 6) using the QUADAS-2 tool. Across most domains, the studies demonstrated a predominance of low risk of bias, particularly within the patient selection and index test domains, indicating generally strong methodological rigor. However, some potential sources of bias were identified: Weikert et al. showed moderate risk in the reference standard domain due to reliance on clinical radiology reports, and Zaazoue et al. raised concerns in the flow and timing domain due to the exclusion of indeterminate cases [19,27]. These isolated concerns are unlikely to substantially impact the overall reliability of the evidence, as the remaining studies (Schmuelling et al., Languis-Wiffen et al., Cheikh et al., and Ayobi et al.) exhibited low risk across all domains [20,25,26,28]. Overall, the low prevalence of high-risk or unclear judgments supports the validity and applicability of the included evidence.

Discussion

This systematic review and meta-analysis assessed the diagnostic performance of FDA-approved machine learning (ML) algorithms for the detection of PE on CTPA. From an initial pool of 431 articles, six retrospective studies met the inclusion criteria, including a total of 9,102 CTPA studies. The pooled sensitivity was 92.5%, and the pooled specificity was 98.2%.

Both included algorithms, Aidoc and CINA-PE, have received FDA clearance, distinguishing this analysis from others that focus on experimental or pre-commercial models. These findings suggest that commercially available ML tools can be both accurate and reliable when deployed in real-world clinical environments.

Diagnostic Accuracy

The performance of these FDA-approved ML models aligns with, and in some cases exceeds, that of experienced radiologists. For instance, Cheikh et al. and Ayobi et al. reported that the ML algorithms outperformed radiologists in sensitivity and in identifying cases missed during routine interpretation [20,26]. Ayobi et al. found that the CINA-PE algorithm reduced the miss rate from 15.6% to 3.8% by identifying 76% of the cases initially overlooked by radiologists, underscoring their potential role as effective safety nets to reduce missed diagnoses [20]. Several studies also demonstrated that ML performance was unaffected by complicating factors such as poor contrast bolus (Cheikh et al.) [26] or the presence of COVID-19-related lung changes (Zaazoue et al.) [27], further supporting the robustness of these tools across a range of clinical scenarios.

Specificity also remained high across studies, with some reporting values nearing 100% [27,28]. This suggests that the algorithms are not only capable of identifying true positive cases but also of minimizing false positives that could otherwise lead to unnecessary downstream testing and anticoagulation.

Workflow Impact

Although diagnostic accuracy was uniformly high, Schmuelling et al. noted no significant improvement in workflow efficiency or clinical endpoints (report communication time, turnaround time, and time to anticoagulation) following ML tool integration [26]. The authors noted that the DL algorithm and electronic notification system were introduced without concurrent changes to standard operating procedures, user training, or structured adoption strategies, which limited their ability to affect broader process outcomes [26]. These factors meant that although the AI system technically functioned, it did not translate into measurable improvements in workflow efficiency metrics, which highlights the importance of considering broader system-level factors - such as radiologist acceptance, integration into Picture Archiving and Communication System (PACS)/Radiology Information System (RIS) workflows, and alert fatigue - when evaluating AI tool impact.

Practical implementation of ML algorithms presents several real-world challenges that can limit successful adoption. Upfront and maintenance costs, the need for structured user training, and the risk of alert fatigue are among the most commonly cited barriers to integration into radiology workflows. Financial constraints may deter smaller or resource-limited institutions from adopting commercial AI tools, given the ongoing costs of software licensing, IT infrastructure, and regulatory compliance [29,30]. Additionally, inadequate training or poor workflow integration can erode radiologists' trust and reduce algorithm utilization [31]. Frequent or nonspecific alerts may also contribute to desensitization and diminished response rates, a phenomenon known as alert fatigue, which has been observed in both radiology and clinical decision support systems [32]. Potential solutions include tailoring AI notification thresholds, embedding decision support directly within PACS to minimize workflow disruption, and incorporating structured training programs that emphasize interpretive synergy between radiologists and AI systems, which are measures that have been proposed to enhance user confidence, reduce cognitive burden, and ensure sustained clinical benefit.

Strengths and Limitations

Overall, the results of this meta-analysis provide strong evidence supporting the diagnostic efficacy of FDA-approved ML algorithms in PE detection, which may justify their broader adoption in clinical practice. Their high diagnostic accuracy, particularly in identifying missed cases, positions them as potential aids in high-volume emergency departments or in settings with radiologist shortages. Also, their demonstrated ability to reduce miss rates positions them as valuable adjuncts in quality assurance and peer-review workflows. However, reliance on these algorithms should not replace radiologist interpretation but rather augment it. For example, these tools may be used as a “second reader” or triage aid that prioritizes suspicious cases for expedited review, enhancing both diagnostic accuracy and efficiency. Institutional policymakers and radiology departments may consider structured integration of these tools to reduce diagnostic errors and improve patient outcomes; however, successful implementation of these algorithms must also account for user training, workflow adaptation, and institutional protocols.

This meta-analysis has several limitations. First, substantial heterogeneity was observed across the included studies (I² = 87.1% for sensitivity, I² = 98.5% for specificity), indicating considerable variability in diagnostic performance estimates. This high degree of heterogeneity means that the pooled sensitivity and specificity values should be interpreted as average effects rather than universally applicable figures, as performance may vary significantly across clinical environments [33]. Consequently, the predictive certainty and generalizability of these pooled estimates are limited, particularly for institutions using different CTPA protocols, scanner types, or patient populations.

Second, although all studies demonstrated a low risk of bias according to QUADAS-2, each was retrospective in design. Prospective, randomized studies remain necessary to evaluate the downstream clinical implications of AI-assisted diagnosis - such as its impact on management decisions, patient outcomes, and cost-effectiveness. The small number of included studies (n = 6) also limited the ability to perform subgroup analyses and may have contributed to the observed heterogeneity. Moreover, five of the six studies evaluated the same algorithm (Aidoc), which may have skewed the pooled estimates and reduced the representativeness of other FDA-cleared tools.

The final limitation is that most included studies were conducted in Western, high-resource healthcare systems, potentially limiting generalizability to lower-resource or non-Western settings where imaging protocols, data quality, and disease prevalence differ. Future research should prioritize geographically diverse and prospective validation studies that assess algorithm performance, workflow integration, and cost-effectiveness in a broader range of clinical environments to establish true global applicability.

Future Directions and Clinical Implications

Prospective trials evaluating the effect of ML-assisted diagnosis on clinical decision-making and patient outcomes remain essential to fully understand the real-world impact of these algorithms on clinical workflow. While retrospective studies demonstrate high diagnostic accuracy, they do not account for practical variables such as workflow integration, radiologist trust, and the potential for alert fatigue. Additionally, cost-effectiveness analyses are needed to assess the feasibility of deploying these tools at scale. Comparative effectiveness studies evaluating different FDA-approved algorithms across diverse clinical settings and patient populations are also warranted to determine their relative strengths and optimal use cases.

Overall, these FDA-approved ML algorithms exhibit high diagnostic performance for detecting PE on CTPA, with pooled sensitivity and specificity exceeding 90% and 98%, respectively. These findings underscore the readiness of such tools for clinical use, provided they are thoughtfully integrated into radiological practice. While promising, future prospective and implementation-focused studies are needed to confirm their real-world impact on patient care.

## Conclusions

This systematic review and meta-analysis demonstrates that FDA-approved ML algorithms for PE detection on CTPA exhibit strong diagnostic performance and hold promise as adjunctive tools in radiology practice, with potential applications in triage, error reduction, and quality assurance. Additionally, these systems may provide added clinical value by reducing diagnostic errors, serving as triage aids in high-volume settings, and supporting quality assurance processes. However, despite their high diagnostic accuracy in retrospective settings, further prospective studies are essential to determine their true clinical impact, including effects on workflow efficiency, treatment timeliness, cost-effectiveness, and patient outcomes. Broader validation across diverse practice environments and direct comparisons between different FDA-approved algorithms will be critical for informing optimal implementation strategies.
